# *SIRT7* deletion inhibits *Glaesserella parasuis*-mediated inflammatory responses in porcine alveolar macrophages

**DOI:** 10.3389/fcimb.2025.1589199

**Published:** 2025-06-25

**Authors:** Hao Zheng, Baoxin Wang, Xia Dong, Junjing Wu, Liangyu Shi, Jing Zhang, Hongbo Chen, Ao Zhou

**Affiliations:** ^1^ Laboratory of Genetic Breeding, Reproduction and Precision Livestock Farming, School of Animal Science and Nutritional Engineering, Wuhan Polytechnic University, Wuhan, China; ^2^ Hubei Provincial Center of Technology Innovation for Domestic Animal Breeding, Wuhan Polytechnic University, Wuhan, China; ^3^ Institute of Animal Science and Veterinary Medicine, Hubei Academy of Agricultural Sciences, Wuhan, China

**Keywords:** SIRT7, *Glaesserella parasuis*, inflammatory responses, CRISPR/Cas9, disease-resistant breeding

## Abstract

*Glaesserella parasuis* (GPS) infection causes severe inflammatory disorder, resulting in lung injury. *SIRT7* is an NAD^+^-dependent deacetylase known to regulate inflammatory responses, but its role in GPS infection remains unclear. Here we found that GPS infection increased *SIRT7* expression and induced inflammatory responses. Deficiency of *SIRT7* by CRISPR/Cas9 technology significantly inhibited GPS-induced cytopathic effects and inflammatory responses. In addition, RNA-seq analysis showed that differentially expressed genes(DEGs) induced by *SIRT7* deficiency were enriched in biological processes such as cell proliferation, actin cytoskeleton formation, lipid synthesis, protein kinase activation regulation, and GTPase activity regulation. Functional enrichment analysis further indicated the involvement of these DEGs in tight junction pathway, PI3K-Akt signaling pathway, actin cytoskeleton regulation, cGMP-PKG signaling pathway, Hippo signaling pathway, and TNF signaling pathway. Finally, we identified some hub genes (*GNAI3*, *GNAI1*, *JAK1*, *NDUFS8*, *CYC1*) related to oxidative phosphorylation. In summary, our results demonstrate that SIRT7 is pivotal for GPS-induced inflammatory responses, which represents a promising target resistant to GPS infection.

## Introduction

The sirtuin family is a class of evolutionarily conserved NAD^+^ (nicotinamide adenine dinucleotide)-dependent deacetylases, with multifunctional roles (Wu et al., 2022). Sirtuins regulate diverse biological processes, such as metabolic regulation, epigenetic modifications, cellular aging, and inflammatory responses (Ji et al., 2022). Among the seven mammalian sirtuins (SIRT1-SIRT7), SIRT1 is predominantly unclear but minor distribution can also localize to the cytosol under certain conditions (Tanno et al., 2007). It is involved in various biological processes, including oxidative stress (Vancura et al., 2018), lipid metabolism (Qiang et al., 2011), apoptosis (Chen et al., 2021), cellular aging (You and Liang, 2023), and inflammation (Yang et al., 2022). SIRT2 is the only predominantly cytoplasmic sirtuin, with minor presence in the nucleus and mitochondria. It plays a crucial physiological role in mammals, being pivotal in aging (Zhang et al., 2023), differentiation (Fang et al., 2019), inflammation (Sola-Sevilla et al., 2023), cancer (Chen et al., 2020), and neurodegenerative diseases (Lu et al., 2023). SIRT3, SIRT4, and SIRT5 localize to mitochondria and are collectively termed mitochondrial sirtuins, key regulators of cellular metabolism (Ji et al., 2022). SIRT6 and SIRT7 are nuclear-localized sirtuins, with SIRT6 enriched in chromatin and SIRT7 residing primarily in the nucleolus (Wu et al., 2022). SIRT6 plays a critical role in regulating cellular energy sensing and homeostasis, linking to cellular aging, metabolism, inflammation, and cardiovascular diseases (Chang et al., 2020; Guo et al., 2022). SIRT7, a recently identified member of the sirtuin family, is involved in maintaining genomic integrity, physiological homeostasis, and anti-aging. SIRT7 deficiency disrupts metabolic homeostasis, accelerates aging, and predisposes to inflammatory disorders, cancer, and cardiovascular diseases (Raza et al., 2024).

The *SIRT7* gene is located on chromosome 12 in the porcine genome and exhibits ubiquitous expression across multiple tissues, including the heart, kidneys, liver, lungs, subcutaneous fat, spleen, and muscles. Its expression is most abundant in the lungs, spleen, and adipose tissue. Studies have revealed that *SIRT7* plays a crucial role in pathogen-host interactions. In HBV-infected cell models, SIRT7 overexpression significantly suppresses HBV RNAs expression, whereas silencing SIRT7 enhances HBV transcription and replication (Yu, 2019). In oral cancer, SIRT7 modulates tumor cell activity by polarizing macrophage phenotypes-suppressing M2 macrophages while activating M1 macrophages, thereby exerting antitumor effects (Gu et al., 2023). Furthermore, the study reveals that SIRT7 plays a protective role in *Mycobacterium tuberculosis* (*Mtb*) infection. SIRT7 functions in combating *Mtb* infection by regulating macrophage nitric oxide (NO) production and apoptosis pathway (Liu, 2020). Recent studies indicate that SIRT7 has an anti-inflammatory role. In lipopolysaccharide (LPS)-stimulated bovine mammary epithelial cells, SIRT7 downregulation exacerbates NF-κB p65 and nuclear translocation, augmenting inflammatory cytokine secretion (Chen et al., 2019). Conversely, SIRT7 silencing attenuates LPS-induced inflammation in lung endothelial cells and reduces renal ischemia-reperfusion injury by suppressing pro-inflammatory cytokines (Wyman et al., 2020; Sánchez-Navarro et al., 2022). Murine colitis models further demonstrate elevated SIRT7 levels in inflamed colonic mucosa, where its knockdown ameliorates inflammation (Kim et al., 2022). Notably, the regulatory function of SIRT7 during *Glaesserella parasuis* (GPS) infection remains unexplored, warranting further investigation.

GPS, a Gram-negative bacterium exhibiting pleomorphic characteristics (including elongated rods, filaments, and coccobacilli), is a common pathogen in swine upper respiratory tracts and the causative agent of Glässer’s disease (Zhang, 2024). It features a capsule, pili, and outer membrane proteins. Based on agar gel diffusion typing, GPS isolates are classified into 15 serotypes, with approximately 20% remaining untypable. Among these, serotypes 1, 5, 10, 12, 13, and 14 demonstrate high pathogenicity, capable of inducing systemic inflammation in piglets. Epidemiological data indicate serotypes 4 and 5 predominate in China, followed by 13, 14, and 12 (Cai et al., 2005). The virulence factors of GPS are numerous, and its pathogenic mechanisms are complex. The specific virulence factors and detailed mechanisms of pathogenicity remain not fully understood (Ye et al., 2021).

RNA sequencing (RNA-seq) is a high-throughput sequencing technique used to sequence and analyze the transcriptome, including both mRNA and noncoding RNAs, from specific cells or tissues. This approach provides unprecedented insights into transcriptional regulation, offering distinct advantages including high sensitivity, broad dynamic range, and strand-specific information. RNA-seq has become indispensable for elucidating molecular mechanisms underlying disease resistance and reproductive traits (Xiu et al., 2023). In this study, to investigate SIRT7’s regulatory role during GPS infection, we first constructed *SIRT7* deficiency 3D4/21 cell lines using CRISPR/Cas9 gene editing technology. RNA-seq analysis comparing wild-type (WT) and SIRT7-knockout (KO) cells enabled systematic identification of differentially expressed genes (DEGs) and associated signaling pathway.

## Materials and methods

### Cell culture and *SIRT7-*knockout 3D4/21 cell lines construction

The Cas9-3D4/21 WT cells and *SIRT7*-KO cell lines were cultured in RPMI 1640 medium (Gibco, USA) containing 10% fetal bovine serum (FBS; Gibco) at 37°C, 5% CO_2_ atmosphere. Cells were passaged every 2–3 days at 90% confluence using 0.25% trypsin-EDTA (Gibco).

*SIRT7-*KO cell lines were generated by using CRISPR/Cas9 gene editing technology. Targeting sites exon 2 and exon 3 of the porcine *SIRT7* gene (ENSSSCG00000034695) were identified using the Ensembl (https://www.ensembl.org/index.html). Two high-score sgRNAs were selected (**Table 1**), and the BbsI restriction endonuclease sticky ends sequences of the pb-U6-puro-BFP vector were added to both ends. PCR primers were designed at both ends of the sgRNA-targeted exons to amplify the DNA sequence of this region. The sgRNA primers (forward and reverse) were then annealed. The vector pb-U6-puro-BFP was digested with the BbsI (NEB, USA). The digested products were recovered using the TIANquick Midi Purification Kit (TianGen, China). The annealed sgRNA duplexes were ligated into BbsI-digested vectors using T4 DNA Ligase (Transgen Biotech, China). The ligation mixture was transformed into competent DH5α cells (TianGen, China) and screened on ampicillin-resistant plates. Single colonies were picked and cultured in LB liquid medium. After expanding the culture, plasmid DNA was extracted using the EndoFree Mini Plasmid Kit II (TianGen, China) and sequenced. The pb-U6-SIRT7-sgRNA-puro-BFP plasmid was transfected into the WT cell lines. After 48 h post-transfection, cells were selected with 1.5 μg/mL puromycin (Beyotime, China) for 7 days until complete death of untransfected controls. When all WT cells died, the surviving cells in the treatment group were further cultured. Surviving single-cell colonies were picked and seeded into 96-well plates for culture. Single-cell colonies were expanded for genotyping by PCR (GoTaq polymerase, Promega) and sequencing. The PCR primers used are listed in **Table 1**.

**Table 1:** Primer sequences.

Gene name	Primer sequence (5,-3,)	Primer purpose
*SIRT7-*sgRNA1	F:CACCGAGGTGCTTGGCGTTGCGGA	Vector construction
	R:AAACTCCGCAACGCCAAGCACCTC	
*SIRT7-*sgRNA2	F:CACCGGTTAGGGCCCCGGTAGTCT	Vector construction
	R:AAACAGACTACCGGGGCCCTAACC	
*SIRT7*	F:GAGAGCGAGGACCTGGTG	PCR
	R:GTGGAAGGGAAGCGGAGG	
*GAPDH*	F:TCGGAGTGAACGGATTTG	qRT-PCR
	R:CCTGGAAGATGGTGATGG	
*IL-6*	F:CCAGGAACCCAGCTATGAAC	qRT-PCR
	R:CTGCACAGCCTCGACATT	
*IL-8*	F:TCTTGGCAGTTTTCCTGCTTT	qRT-PCR
	R:AATTTGGGGTGGAAAGGTGT	
*TNF-α*	F:GCTCTTCTGCCTACTGCACTTC	qRT-PCR
	R:GTCCCTCGGCTTTGACATT	

### Bacteria culture

GPS serotype 5 strain (SH0165) was provided by the *Laboratory of Genetic Breeding Reproduction and Precision Farming, School of Animal Science and Nutrition Engineering, Wuhan Polytechnic University*. Frozen stock cultures were rapidly thawed at 37°C and inoculated into Tryptic Soy Broth (TSB; BD, USA) supplemented with 10% FBS and 0.01% NAD (Beyotime), and cultured at 37°C with 180 rpm shaking for 10–12 h. The medium was then checked for turbidity. Subsequently, the culture was streaked onto TSA (BD) plates and incubated at 37°C for 36 h.

### Genomic identification of the *SIRT7*

Genomic DNA was isolated from *SIRT7*-KO cell lines using the Universal Genomic DNA Kit (CWBIO, China) and amplified by PCR and sequenced. The PCR products were analyzed by agarose gel electrophoresis. Based on the size of the electrophoresis bands, gene deletions or insertions were assessed. PCR products were subjected to gel extraction (TIANGEN, China) and sent to Sangon Biotech for sequencing. The sequencing results were compared with WT cell sequences to further verify if fragment deletions occurred between the designed sgRNA target sites.

### Quantitative real-time PCR

Cells were collected at different time points post-infection, and total RNA was extracted using RNAsimple Total RNA Kit (TIANGEN) according to the manufacturer’s instructions. Following RNA quality assessment, RNA samples were reverse-transcribed into cDNA using HiScript II Q RT SuperMix (Vazyme, China). The relative mRNA levels were quantified by qRT-PCR and calculated via the 2^-ΔΔCT^ method with normalization to the internal control gene *GAPDH.* The amplification protocol consisted of 95°C for 30 s, 40 cycles of 95°C for 15 s and 60°C for 1 min. Primer sequences are listed in **Table 1**.

### Western blotting

Proteins were extracted from GPS-infected cells at different time points, and the protein concentrations were determined using BCA Protein Quantification Kit (Vazyme, China). Protein samples were separated by SDS-PAGE electrophoresis and transferred to PVDF membranes. The membranes were blocked with 5% non-fat milk and subsequently incubated with the primary antibodies [GAPDH (Proteintech, USA) and SIRT7 (FineTest, China)] overnight at 4°C followed by incubation with the secondary antibody at room temperature for 1 h. After three washes with TBST buffer, protein signals were detected using SuperPico ECL Chemiluminescence Kit (Vazyme) with a chemiluminescence imaging system.

### RNA-seq analysis, DEGs identification and functional enrichment analysis

RNA-Seq technology was performed to identify the DEGs in *SIRT7*-KO cells. Total RNA was extracted using the TRIzol method for RNA-seq (Zhenyue Biotechnology, China) with the MGI DNBseq-T7 platform (BGI, Hong Kong) with 150 bp paired-end reads. Raw reads from RNA-seq libraries were filtered to obtain clean reads, which were then aligned to *Sus scrofa* reference genome (Sscrofa11.1) using HISAT2 with default parameters. DEGs were identified using DESeq2 (v1.38.3) with the thresholds of *P* < 0.05 and |log_2_ Foldchange| > 1. Functional enrichment analysis was conducted through Gene Ontology (GO) and Kyoto Encyclopedia of Genes and Genomes (KEGG) pathway analyses using the clusterProfiler R package. Protein-protein interaction (PPI) networks were constructed using the STRING database, and hub genes were identified via the cytoHubba plugin (v0.1) in Cytoscape software.

## Results

### SIRT7 expression was upregulated in GPS-infected porcine alveolar macrophages

**Figure 1:**
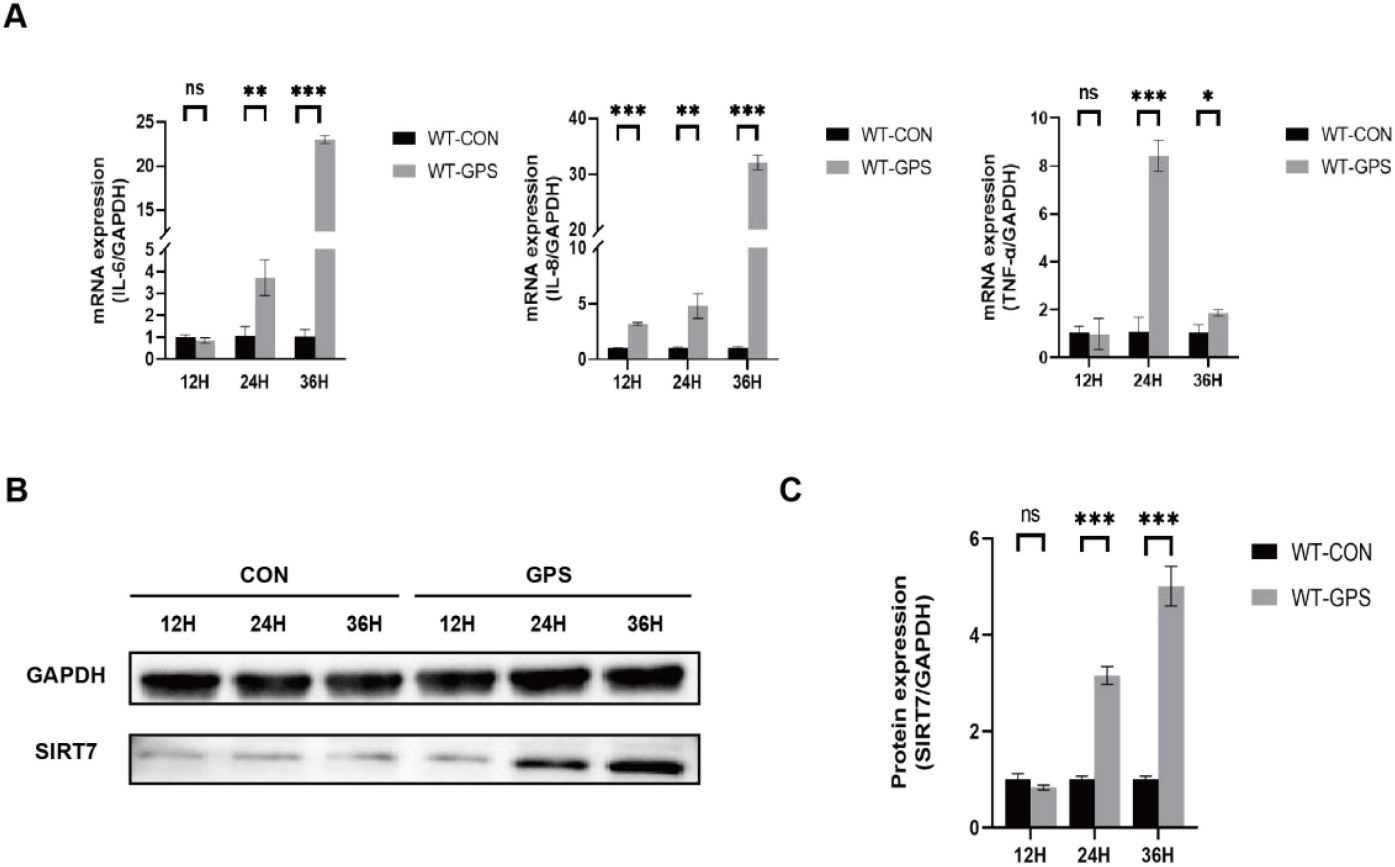
SIRT7 is involved in GPS-induced inflammatory response. **(A)** Relative expression levels of inflammatory factor genes after GPS infection in WT cells. **(B)** Expression levels of SIRT7 protein after GPS infection in WT cells. **(C)** Quantitative analysis of SIRT7/GAPDH gray values. Statistical significance versus control: *P < 0.05, **P < 0.01, ***P < 0.001.

To analyze the role of SIRT7 in GPS infection, we first detected the expression of SIRT7 in 3D4/21 cells (WT cells) with GPS infection at MOI of 10. QRT-PCR analysis revealed significant upregulation of pro-inflammatory cytokines (*IL-6*, *IL-8* and *TNF-α*) in GPS-infected cells compared to uninfected controls, demonstrating that the inflammatory model of GPS-infected cells has been successfully established (**Figure 1A**). Western blot analysis found that the protein expression of SIRT7 was significantly upregulated during GPS infection (**Figures 1B, C**), indicating that SIRT7 may participate in GPS-induced inflammatory response.

### Construction of *SIRT7*-KO cell line

**Figure 2:**
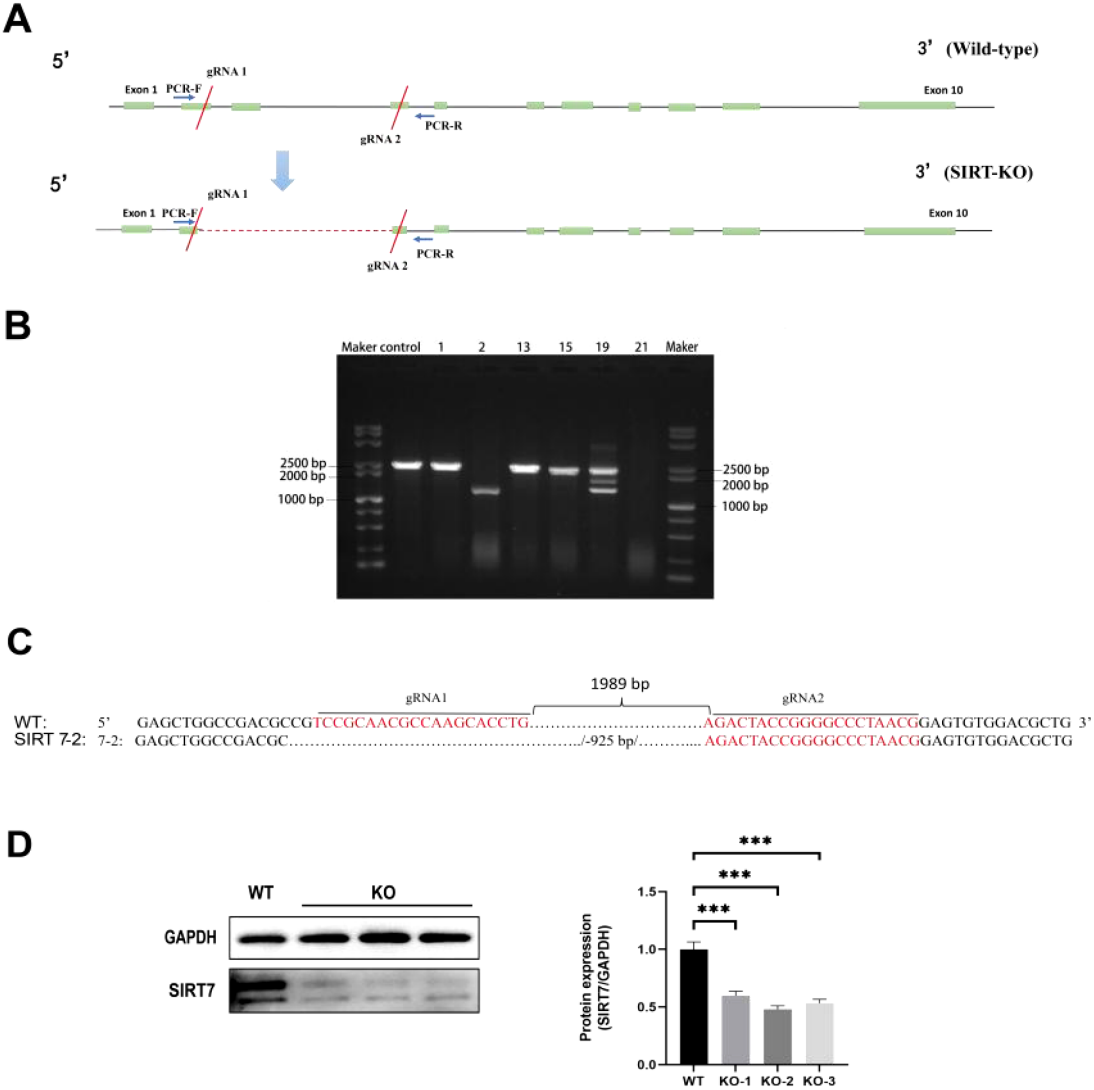
Construction of *SIRT7*-KO cell line. **(A)** *SIRT7* gene structure and gRNA targeting sites. **(B)** PCR analysis (M: DL100 DNA Marker; Control: WT cells; 1, 2, 13, 15, 19, 21: Monoclonal cell lines). **(C)** The sequencing results of *SIRT7*-KO. **(D)** SIRT7 protein expression assessment in the KO cell line (internal control: GAPDH) included three biological replicates: KO-1, KO-1, KO-3. Data represent mean ± standard deviation (n = 3). Statistical significance versus control: ***P < 0.001.

*SIRT7*-KO cell line was established by CRISPR/Cas9 system (**Figure 2A**). After picking the single cell colonies, DNA was extracted and amplified by PCR. Based on the electrophoresis and sequencing results, we found that clone 2 amplified a 1408 bp band and had a mutation with a 925-bp deletion near the protospacer adjacent motif (PAM) sequence, while the WT sample had a 2333 bp band (**Figures 2B, C**). Western blot analysis showed that SIRT7 protein expression was significantly reduced in KO cell line compared with WT cells (**Figure 2D**).

### SIRT7 deficiency reduced GPS-induced cell damage and inflammatory responses

**Figure 3:**
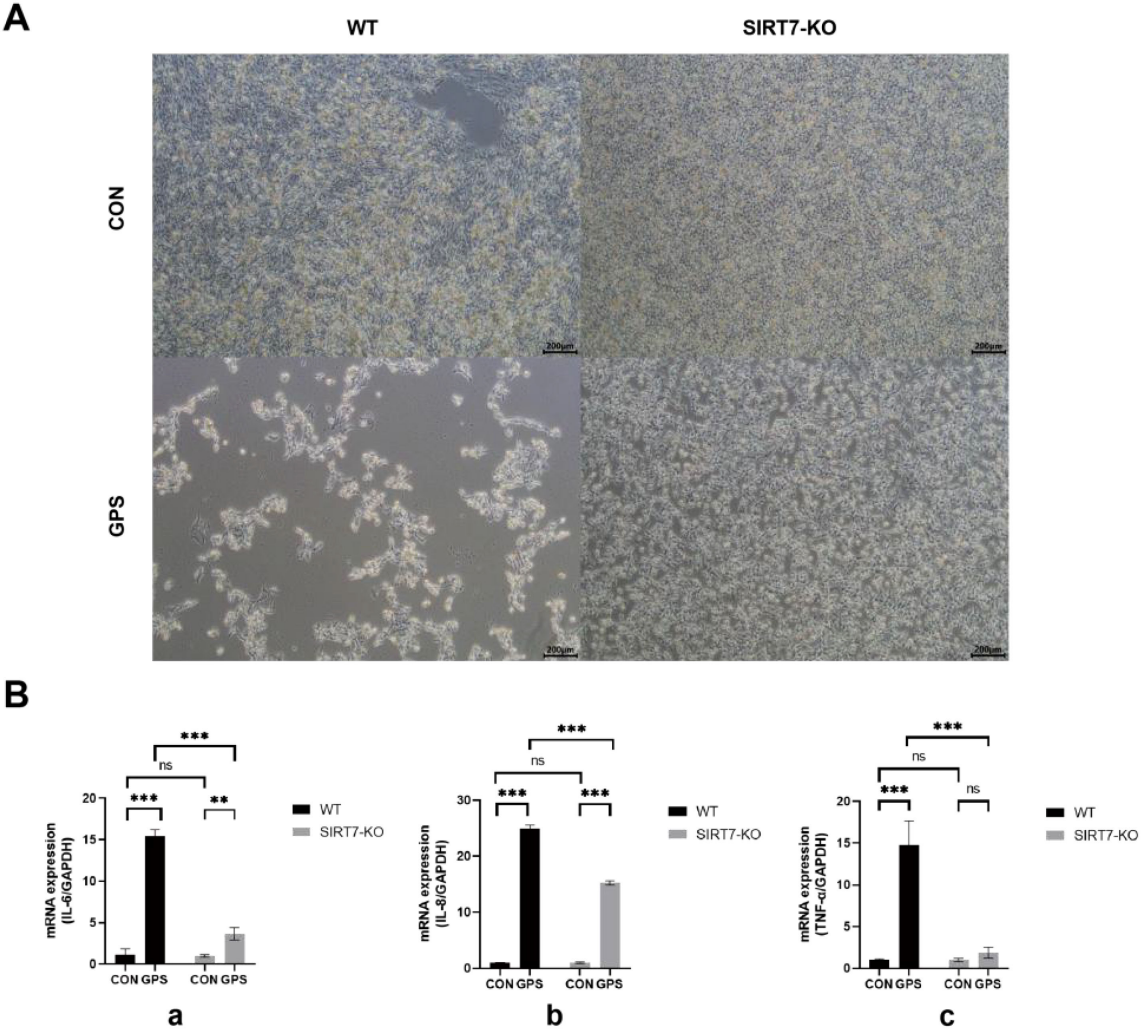
**(A)** *SIRT7* deficiency inhibits GPS-induced cellular damage. **(B)** *SIRT7* deficiency inhibits the inflammatory responses induced by GPS infection. (a: *IL-6* expression levels; b: *IL-8* expression levels; c: *TNF-α* expression levels). Data represent mean ± standard deviation (n = 3). Statistical significance versus control: *P < 0.05, **P < 0.01, ***P < 0.001.

To analyze the function of SIRT7 in GPS infection, WT and *SIRT7*-KO cells were infected with GPS at MOI=10. The results indicated that *SIRT7* deficiency significantly ameliorated GPS-induced cytopathic effects compared to the WT cells (**Figure 3A**). In addition, pro-inflammatory cytokine mRNA expression (*IL-6*, *IL-8*, *TNF-α*) were significantly downregulated in *SIRT7*-KO cells compared to the WT cells after GPS treatment (**Figure 3B**). These results indicated that the SIRT7 might plays a pivotal role in mediating inflammatory responses triggered by GPS infection.

### DEGs identification and function enrichment analysis in *SIRT7-*KO cells

**Figure 4:**
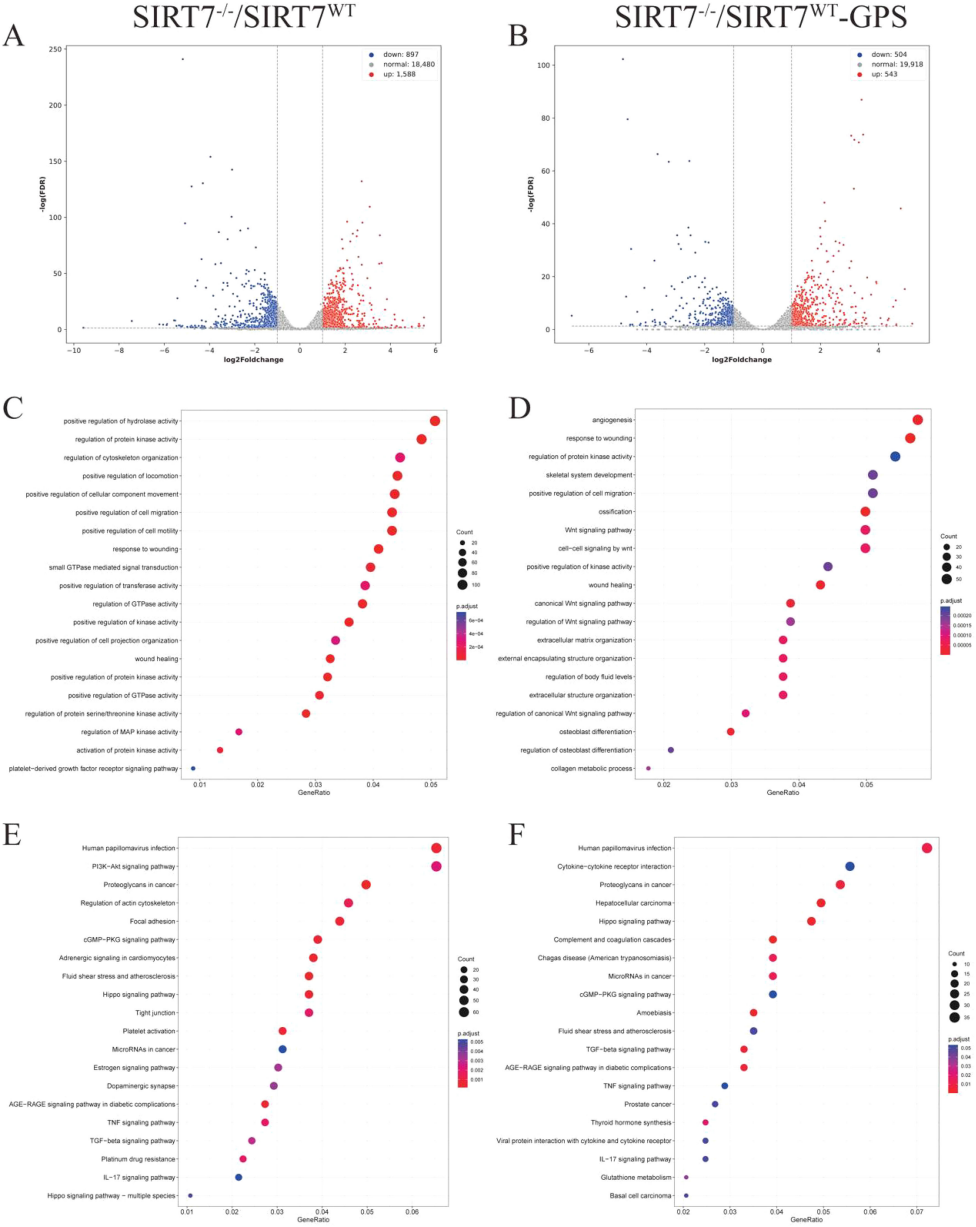
SIRT7 deficiency affects the transcriptome regulation. **(A)** Volcano plots of DEGs in *SIRT7-*KO cells without GPS infection. **(B)** Volcano plots of DEGs in *SIRT7*-KO cells with GPS infection. **(C, D)** GO enrichment analysis. **(E, F)** KEGG function enrichment analysis.

To elucidate the molecular mechanisms by which SIRT7 regulates GPS infection, RNA-Seq was used to detect the mRNA expression profiles of *SIRT7*-KO cells and WT cells with or without GPS infection. 1,588 upregulated and 897 downregulated DEGs were identified in *SIRT7*-KO cells compared to their expressions without GPS infection using the selection criteria: |log_2_Foldchange| ≥ 1 and *P* < 0.05 (**Figure 4A**, and **Supplementary Table S1**), While 543 genes were significantly upregulated, and 504 genes were significantly downregulated in *SIRT7*-KO cells compared to their expressions in WT cells infected with GPS (**Figure 4B**, and **Supplementary Table S2**). Furthermore, functional enrichment analysis showed that protein processing associated terms were significantly enriched with or without GPS infection, but some of those DEGs without infection were mainly associated with regulation of GTPase activity, regulation of cytoskeleton organization, while the biological process of some of those DEGs with infection was enriched in angiogenesis, ossification and Wnt signaling pathway (**Figures 4C, D**). In addition, we observed enriched immune response related terms such as cGMP-PKG signaling pathway, TNF signaling pathway and IL-17 signaling pathway, as well as human papillomavirus infection in *SIRT7*-KO cells with or without GPS infection. Notably, PI3K-Akt signaling and tight junction pathway showed specific enrichment in *SIRT7*-KO cells without infection compared to WT cells (**Figures 4E, F**).

### Identification of key gene clusters regulated by SIRT7 deficiency in response to GPS infection

**Figure 5:**
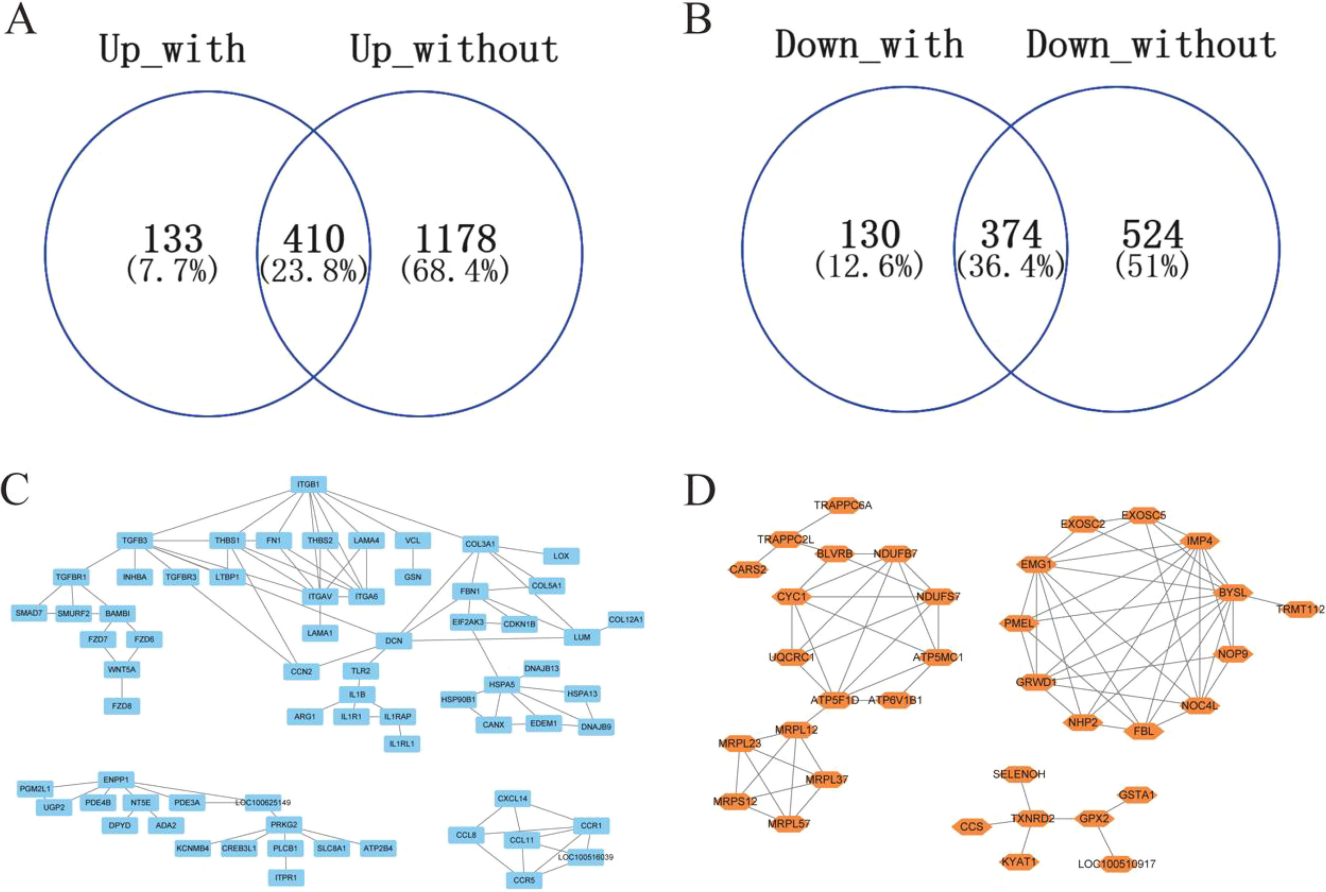
Identification of key gene clusters regulated by SIRT7 deficiency. **(A)** The overlapping upregulated DEGs in SIRT7 deficiency between control and GPS infection. **(B)** The overlapping upregulated DEGs in SIRT7 deficiency between control and GPS infection. **(C, D)** The k-means clustering of the overlapping DEGs in SIRT7 deficiency.

To systematically characterize SIRT7-regulated key gene clusters during GPS infection, we identified overlapping DEGs between *SIRT7*-KO and WT cells under both basal and infected conditions (**Figures 5A, B**; **Supplementary Table S3**). We then performed *k*-means clustering on the upregulated and downregulated DEGs, respectively.

We found that the upregulated common DEGs were divided into three clusters based on their expression patterns, which were mainly associated with extracellular matrix organization, cGMP-PKG signaling pathway, inflammation and immune response (**Figure 5C**), while three clusters were also identified in the downregulated common DEGs and were enriched in functions associated with oxidative phosphorylation, ribosome biogenesis, glutathione metabolism (**Figure 5D**). In addition, we used the cytohubba plugin in Cytoscape software to identify the upregulated hub genes (*ITGB1, ITGAV, ITGA6, THBS1, FN1, TGFB3, CCR1, FBN1, COL3A1, HSPA5*) and the downregulated hub genes (*NDUFS7*, *NDUFB7*, *BYSL, IMP4, EMG1, GRWD1, NOC4L, FBL, ATP5F1D, CYC1*), which might coregulate GPS infection with SIRT7.

## Discussion

GPS is a non-motile, encapsulated, facultatively anaerobic Gram-negative bacterium that lacks spore-forming capacity and hemolytic activity (Brockmeier et al., 2014). GPS is the etiological agent of Glässer’s disease, characterized by multiple fibrinous pleuritis, arthritis, and meningitis (Sun, 2024). As a commensal-turned-pathogen, GPS colonizes the porcine upper respiratory tract and becomes invasive under immunosuppressive conditions, such as viral co-infections (e.g., PRRSV) or stress-induced immunocompromise. With the intensification of pig farming and widespread antibiotic misuse, the prevalence of GPS has been rising in China, resulting in significant economic losses to the pig industry (Dai et al., 2024). Research indicates that GPS infection induced cellular inflammation and damage (Zeng et al., 2022). In this study, we observed significant upregulation of *IL-6, IL-8*, and *TNF-α* mRNA levels, confirming the successful establishment of a GPS infection model in 3D4/21 cells.

As a nuclear-localized member of the NAD^+^-dependent sirtuin deacetylase family, SIRT7 has emerged as a key epigenetic regulator in host-pathogen interactions. SIRT7 decreases IRF3/IRF7 phosphorylation to block the interaction between tbk1 and IRF3/IRF7, resulting in the suppression of antiviral responses, disruption of SIRT7 increases the survival rate of carp during virus infection (Liao et al., 2021). Newcastle disease virus infection induced *SIRT7* expression, which in turn enhances cellular proteins deacetylation causing high virus replication (Shokeen and Kumar, 2024).However, the regulatory mechanism of the *SIRT7* gene in GPS infection has not yet been reported. Our results showed that GPS infection upregulates SIRT7 expression in macrophages suggests its potential involvement in antibacterial defense mechanisms.

This study established a model by creating *SIRT7*-KO cell lines with CRISPR/Cas9 technology. It was found that *SIRT7*-KO cells showed less cell damage infection, and significantly reduced expression of pro-inflammatory cytokines (*IL-6*, *IL-8*, *TNF-α*) upon GPS infection, suggesting that SIRT7 deficiency can suppress the occurrence of inflammation induced by GPS infection. Previous studies reported that SIRT7 played a key role in regulating the inflammatory responses, and silencing SIRT7 can inhibit LPS-induced pro-inflammatory responses and NF-κB signaling (Miyasato et al., 2018; Wyman et al., 2020; Mizumoto et al., 2022; Mizutani et al., 2022; Sánchez-Navarro et al., 2022). RNA-seq results revealed that the DEGs induced by *SIRT7* deficiency were mainly enriched in biological processes such as cell proliferation, actin cytoskeleton organization, lipid synthesis, regulation of protein kinase activity, and GTPase activity. KEGG pathway analysis identified enrichment in pathways related to viral infection, cancer, tight junctions, PI3K-Akt signaling, actin cytoskeleton regulation, cGMP-PKG signaling, Hippo signaling, and TNF signaling, suggesting that SIRT7 may regulate GPS infection via these signaling pathways. The Hippo signaling pathway plays a crucial role in regulating many biological processes, such as cell proliferation, differentiation, and stem cell self-renewal (Kwon et al., 2013). Dysregulation of the Hippo signaling pathway can lead to various diseases (Fu et al., 2022). The Hippo signaling pathway regulates lung inflammation, with increased activity of type II alveolar epithelial cells associated with elevated nuclear expression of Hippo signaling mediators Yap and Taz. The absence of Yap/Taz in mice affects the repair and regeneration of alveolar epithelial cells in bacterial pneumonia, while downregulation of YAP1 alleviates LPS-induced lung injury by promoting M2 macrophage polarization (Lacanna et al., 2019; Liang et al., 2023). Additionally, the *SIRT7* gene activates the Hippo/YAP signaling pathway by directly binding to MST1 and deacetylating it, leading to MST1 ubiquitination and degradation (Gu et al., 2024).

The PI3K/Akt axis plays a crucial role in recruiting and activating innate immune cells, such as macrophages, while exhibiting dual regulatory effects on inflammatory factors production (Hawkins and Stephens, 2015). Studies indicate that under chronic overnutrition conditions, elecated pro-inflammatory factors activate mTORC1, which subsequently suppresses the PI3K/Akt signaling pathway through Ser/Thr residue phosphorylation (Saxton and Sabatini, 2017). Notably, extensive research shows that the sirtuin family can enhance autophagy by inhibiting the PI3K/Akt signaling pathway (Yang et al., 2019; Mishra et al., 2022; Zhang et al., 2022; Zhou et al., 2023). We hypothesize that SIRT7 may enhance autophagy by inhibiting the PI3K/Akt pathway, thereby exacerbating inflammatory responses.

The tight junction signaling pathway maintains tissue homeostasis and integrity by regulating paracellular permeability and cell polarity. Tight junction proteins play a crucial role in regulating cell proliferation, migration, and differentiation (Nehme et al., 2023). Research indicates that overexpression of SIRT6 can restore the expression of tight junction proteins and alleviate cell apoptosis and inflammatory responses (Liu et al., 2023). SIRT1-activated by quercetin upregulates tight junction proteins to mitigate LPS-induced alveolar damage (Deng et al., 2023). Additionally, curcumin activates the AMPK/SIRT3/SOD2/mtROS axis, significantly downregulating inflammatory factors expression levels and increasing tight junction proteins expression (Xiao et al., 2023). We propose that SIRT7 deficiency activates tight-junction signaling, thereby attenuating GPS-induced inflammation. The *k*-means clustering analysis further revealed the expression pattern shift of key gene clusters regulating GPS infection is due to SIRT7 deficiency. All of the upregulated hub genes (*ITGB1, ITGAV, ITGA6, THBS1, FN1, TGFB3, CCR1, FBN1, COL3A1, HSPA5*) regulate inflammation. Overexpression of ITGB1 can reduce LPS-induced inflammation and oxidative stress (Qi et al., 2024). Knockdown of THBS1 inhibits autophagy and promotes NLRP3 inflammasome formation (Chiu et al., 2024). HSPA5 can be deacetylated by inhibition of SIRT1/2 to trigger the pro-survival autophagy, suggesting that SIRT7 may also interact with HSPA5 to regulate GPS-induced inflammation response (Mu et al., 2019). Moreover, *BYSL, EMG1, GRWD1, FBL* and *NOC4L* were identified as the downregulated hub genes associated with ribosome biogenesis, a process known to suppress immune response (Gratenstein et al., 2005; Adachi et al., 2007; Schilling et al., 2012; Bianco and Mohr, 2019; El Hassouni et al., 2019; Zhu et al., 2019). This suggests that SIRT7 may interact with these hub genes to regulate inflammation response or ribosome biogenesis for affecting GPS infection.

## Conclusion

In our study, SIRT7 deficiency significantly suppressed cytopathy and inflammation induced by GPS infection. Combining with RNA-seq, we found that SIRT7 deficiency alters the expression pattern of gene related to tight junctions, ribosome biogenesis, cGMP-PKG signaling pathway, TNF signaling pathway and IL-17 signaling pathway, regulating GPS-induced inflammatory response. Our results provide possible implications of SIRT7 inhibition as a therapeutic strategy against GPS infection.

## Data Availability

All data utilized in this manuscript are available online from their respective databases. The raw data has been deposited in https://doi.org/10.57760/sciencedb.26878.
